# Case‐Based Immunology: B Cells and Systemic Sclerosis Interstitial Lung Disease

**DOI:** 10.1002/art.43326

**Published:** 2025-12-02

**Authors:** Nina Goldman, Voon Ong, Christopher P. Denton

**Affiliations:** ^1^ Centre for Rheumatology, Division of Medicine University College London London United Kingdom

## Abstract

Interstitial lung disease (ILD) is an important complication of systemic sclerosis (SSc), with high mortality and morbidity. Recent clinical studies in SSc‐ILD have led to US Food and Drug Administration–approved therapies in SSc‐ILD. Importantly, evidence from these studies has been extrapolated to guide management of ILDs of other systemic autoimmune rheumatic diseases. Pathogenesis of SSc‐ILD involves interplay between fibroblasts and the innate and adaptive immune system. A central role for the B cell compartment is supported by clinical and translational studies. We use a case from our center as a basis to discuss the pathogenesis of SSc‐ILD, autoantibodies in SSc‐ILD, and the role of B cells in the disease. We go on to consider treatment options for the case, the decision‐making algorithm for treatment, and risks associated with treatment.

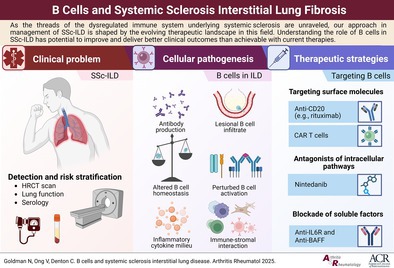

## The clinical case

The patient, a 69‐year‐old man diagnosed with diffuse cutaneous systemic sclerosis^1^ (dcSSc) in 2014, returned for clinic review in May 2022. He reported increasing dyspnea and worsening exercise tolerance over the preceding year without significant cough, chest pain, or ankle edema. He has had recurrent urinary tract infections requiring multiple antibiotic courses but no documented chest infection.

A history review revealed first presentation with Raynaud phenomenon symptoms in September 2013, followed by digital puffiness in January 2014 with progressive skin thickening over the upper limbs and anterior chest over the next six months. He reported reflux, proximal muscle weakness, and weight loss of 6 kg over a similar interval. He was referred to his local rheumatologist and was diagnosed with dcSSc in August 2014. The modified Rodnan skin score (mRSS) peaked at 19/51 in September 2014. Initial investigations at disease presentation in August 2014 showed a hemoglobin level of 127 g/L, a creatine kinase level of 757 IU, a C‐reactive protein (CRP) level of <1 mg/L, an erythrocyte sedimentation rate (ESR) of 2 mm/hr, and an N‐terminal pro–brain natriuretic peptide (NT‐proBNP) level of 26 pmol/L (high >236 pmol/L). Esophageal dysmotility was confirmed with pH manometry and motility studies. Interstitial lung disease (ILD) was confirmed on a high‐resolution computed tomography (HRCT) scan at the time of diagnosis.

In August 2014, he was started on mycophenolate mofetil (MMF) 2 g daily for active skin disease and ILD, with prednisolone 20 mg daily for myositis. In late 2014, he developed complete heart block necessitating permanent pacemaker implantation. A follow‐up echocardiogram in 2015 showed a left ventricular ejection fraction of 45% and regional wall motion abnormality with slight hypokinesia of the left ventricular apex.

His maintenance immunosuppressive therapies comprise MMF 2 g and prednisolone 7.5 mg daily. At the clinic review, the patient reports ongoing SSc gastrointestinal involvement alongside his respiratory symptoms. Despite high‐dose proton pump inhibitors and H_2_ receptor antagonists for gastroesophageal reflux disease, prokinetics for esophageal dysmotility, and rotational antibiotics for small intestinal bacterial overgrowth, he remained symptomatic with vomiting, diarrhea, and bloating. His weight was maintained with high‐calorie supplements.

On examination, the mRSS was stable at 9/51. Chest auscultation demonstrated fine bilateral inspiratory crackles. Autoimmune serology demonstrated speckled‐pattern antinuclear autoantibodies (ANAs) at a titer of 1:1,280 and negative extractable nuclear antigens, with no SSc‐specific antibody identified by standard immunoblot. Available laboratory test results included a hemoglobin level of 154 g/L, a CRP level of 2 mg/L, and an ESR of 5 mm/hr. Cardiac markers performed included the following results: an NT‐proBNP level of 230 ng/L and a troponin T level of 19 ng/L. The recent echocardiogram demonstrated a low probability of pulmonary hypertension.

## Case progress

Results of a repeat lung function test are shown in Supplemental Table 1 and Supplemental Figure 1. The lung function test showed forced vital capacity (FVC) and diffusing capacity for carbon monoxide (DLco) decline of 18.2% and 11.8%, respectively, over the preceding year from May 2021 to May 2022.

This case highlights development of SSc‐ILD and provides a platform for discussion of recent progress in understanding pathogenesis, risk stratification, and treatment options for this complication, which is one of the leading causes of mortality and morbidity in SSc.^2^, ^3^ Informed consent was obtained for use of the clinical information from the patient for publication.

## Pathogenesis of SSc‐ILD

It is hypothesized that initiation of SSc‐ILD involves both endothelial and epithelial cell damage triggered by environmental factors in a susceptible individual. This cellular damage results in the release of locally active mediators such as endothelin 1 and chemokines that may precede activation of the immune system.^4^ Immune system activation then results in an inflammatory cell infiltrate including B cells, macrophages, T cells, and natural killer cells and profibrotic pathway activation, particularly transforming growth factor β (TGFβ) pathways. Subsequently, fibroblasts are activated locally; transdifferentiate from other cell lineages, including epithelial cells and vascular components such as pericytes and endothelial cells^5^; and are recruited and differentiate into profibrotic cells producing excessive extracellular matrix, resulting in deposition of the extracellular matrix within the lung parenchyma and further TGFβ release.^6^ This perpetuates the cycle of lung damage alongside ongoing inflammation and abnormal healing. Other factors such as infection, aspiration, and increased lung stiffness also play a role in exacerbating lung damage.

Genetic and epigenetics changes have been demonstrated to influence an individual's susceptibility to SSc‐ILD, with HLA‐related and non–HLA‐related genetic polymorphisms implicated. The strongest genetic association with ILD is linked to ANA subgroup. Studies have confirmed that ANA reactivity links closely to major histocompatibility complex (MHC) class II haplotype, with HLA‐DRB1*11 conferring considerable risk in patients who are positive for anti–topoisomerase I antibodies (ATAs).^7^

Many risk factors for development and progression of SSc‐ILD have been identified. Patients with dcSSc are at higher risk of clinically significant ILD compared to patients with limited cutaneous SSc (lcSSc).^8^ Other risk factors for progressive disease include early disease duration, male sex, African American race, autoantibody subset, reflux, low baseline FVC and DLco, CT extent above 20%, and FVC decline of at least 10% from baseline.^9^ Elevated levels of interleukin‐6 (IL‐6), the proinflammatory pleiotropic cytokine, have also been shown to be predictive of progression of early ILD, and high CRP levels have also been associated with worse SSc‐ILD outcome.^10^

## Understanding the autoantibody profile

The presence of ANAs is a hallmark of SSc, found in more than 95% of patients with SSc.^11^ Due to their mutual exclusivity, likely a consequence of MHC restriction,^12^ ANA specificities have emerged as central in helping to stratify the risk of organ complications for patients, with the most frequent reactivities being anticentromere antibody (ACA), ATA, and anti–RNA polymerase III antibody (anti–RNAP III), found in >50% of patients with SSc.^11^, ^13^

The focus in SSc‐ILD remains on ATA‐positive patients, with ATA positivity resulting in a much higher risk of clinically significant ILD than low‐risk ACA and intermediate‐risk anti–RNAP III.^8^ However, other SSc‐specific autoantibodies, including anti‐Th/To, anti–eukaryotic initiation factor 2b (anti‐eIF2b), and anti–U11/U12 RNP, and SSc overlap–associated antibodies anti‐PM/Scl and anti‐Ku are also associated with a high risk of SSc‐ILD. Other novel autoantibodies, including anti–nucleolar organizer region antibodies and anti–Bicaudal D protein antibodies, have been variably associated with ILD^14^, ^15^, ^16^ (Table 1). The ANA immunofluorescence pattern can be of assistance, particularly with autoantibodies not routinely tested in clinical practice, with anti‐PM/Scl and anti‐Th/To resulting in a nucleolar pattern, anti–U11/12 RNP (also known as anti‐RNPC3) and anti‐Ku resulting in a speckled pattern, and anti‐eIF2b resulting in a cytoplasmic pattern.^17^ The American College of Rheumatology (ACR) and the American College of Chest Physicians (CHEST) guidelines list ATA positivity and ANA with a nucleolar pattern as risk antibodies for progressive SSc‐ILD; however, other rarer high‐risk antibodies are being defined and should be considered in SSc‐ILD screening.^18^

**Table 1:** Main associations of autoantibody profile in scleroderma‐associated ILD*

Antibody	ANA pattern	Intracellular target	Prevalence in SSc cohort	Cutaneous subset association	Lung involvement	Comments
ATA	Homogeneous or nucleolar/speckled	Type I topoisomerase	20%–30%	Diffuse in 60%	80% develop ILD, of which up to 50% may be progressive	Prognostic for lung function decline irrespective of skin subset; predictive of response to tocilizumab
anti–RNAP III	Nucleolar/homogenous	RNA polymerase type 3	4%–20%	Diffuse phenotype with early severe skin disease followed by potential for rapid spontaneous resolution	Propensity for late lung function decline	–
Anti‐Th/To	Nucleolar	Nucleolar 7‐2/8‐2 RNA protein complex	2%–6%	Limited or sine SSc	50% develop, of which 30% progress	Coexisting pulmonary hypertension may occur
Anti‐U11/U12	Speckled	U11/U12 RNA polymerase	1%–3%	Limited/diffuse	80% develop often severe and progressive ILD	Coexisting severe gut involvement with increased risk of cancer
Anti‐PM‐Scl	Nucleolar	Nucleolar PM/Scl macromolecular complex	3%–6% (25% of SSc–myositis overlap)	Limited or sine SSc	35%–87% develop with good outcome	Organizing pneumonia pattern may occur in context of myositis overlap phenotype
Anti‐Ku	Speckled	Ku complex (p70/p80 heterodimer)	2%–7% (15% of SSc–myositis overlap)	Limited	Up to 76% develop ILD with good outcome	–
Anti–U1 RNP	Coarse speckled	Small nuclear ribonucleoproteins	5%–35% (100% in MCTD)	Limited	35% develop, of which 20% progress	–
Anti‐eIF2B	ANA‐negative with cytoplasmic staining	Eukaryotic initiation factor 2b	1%–2%	Diffuse	High incidence and up to 100% develop	Possible malignancy association
Anti‐RuvBL1/2	Speckled	RuvBL1/2 double hexamer	1%–2%	Diffuse and myositis overlap	Higher frequency in men and higher age at onset with myositis, including cardiac involvement and gut dysmotility	–
Anti‐RNPC3	Speckled	Small nuclear ribonucleoproteins U11 and U12	3%–5%	Diffuse and limited	Coexisting PAH and GI disease and myositis and malignancy	–
Anti‐BICD2	Speckled and/or nucleolar?	Bicaudal D protein	20%–35%	Diffuse	Associated myositis, may coexist with ACA	–
Anti‐NOR90	Nucleolar punctate		2%–3%	Limited	Good prognosis	Variably associated with increased risk of ILD
Anti‐PRMT5	Enzyme belonging to arginine methyltransferases	Unknown	31%	Diffuse	May coexist with ATA	–
Anti‐TERF1	Unknown		9%	Limited and diffuse	May occur with anti‐Ku and anti–U1 RNP	–

* ACA, anticentromere antibody; ANA, antinuclear autoantibody; anti‐eIF2b, anti–eukaryotic initiation factor 2b; anti–RNAP III, anti–RNA polymerase III antibody; ATA, anti–topoisomerase I antibody; GI, gastrointestinal; ILD, interstitial lung disease; MCTD, mixed connective tissue disease; PAH, pulmonary arterial hypertension; SSc, systemic sclerosis.

Anti–U11/U12 RNP has been found in 3% to 8% of patients with SSc.^19^, ^20^, ^21^, ^22^ U11 and U12 are components of the spliceosome and are involved in splicing of pre–messenger RNA.^23^ U11/U12 autoantibodies that react with the 65‐kDa protein component in the U11/U12 small nuclear RNP particle complex were first reported in a patient with SSc by Gilliam and Steitz^24^ and occurs in both skin subtypes of SSc and has been associated with severe ILD.^19^, ^21^ Patients with this antibody are more likely to be male and have moderate to severe gastrointestinal involvement.^25^ In one study, anti–U11/U12 RNP has also been associated with malignancy, with a short interval between SSc diagnosis and cancer diagnosis, although this was not replicated in a subsequent study.^22^, ^26^

**Figure 1:**
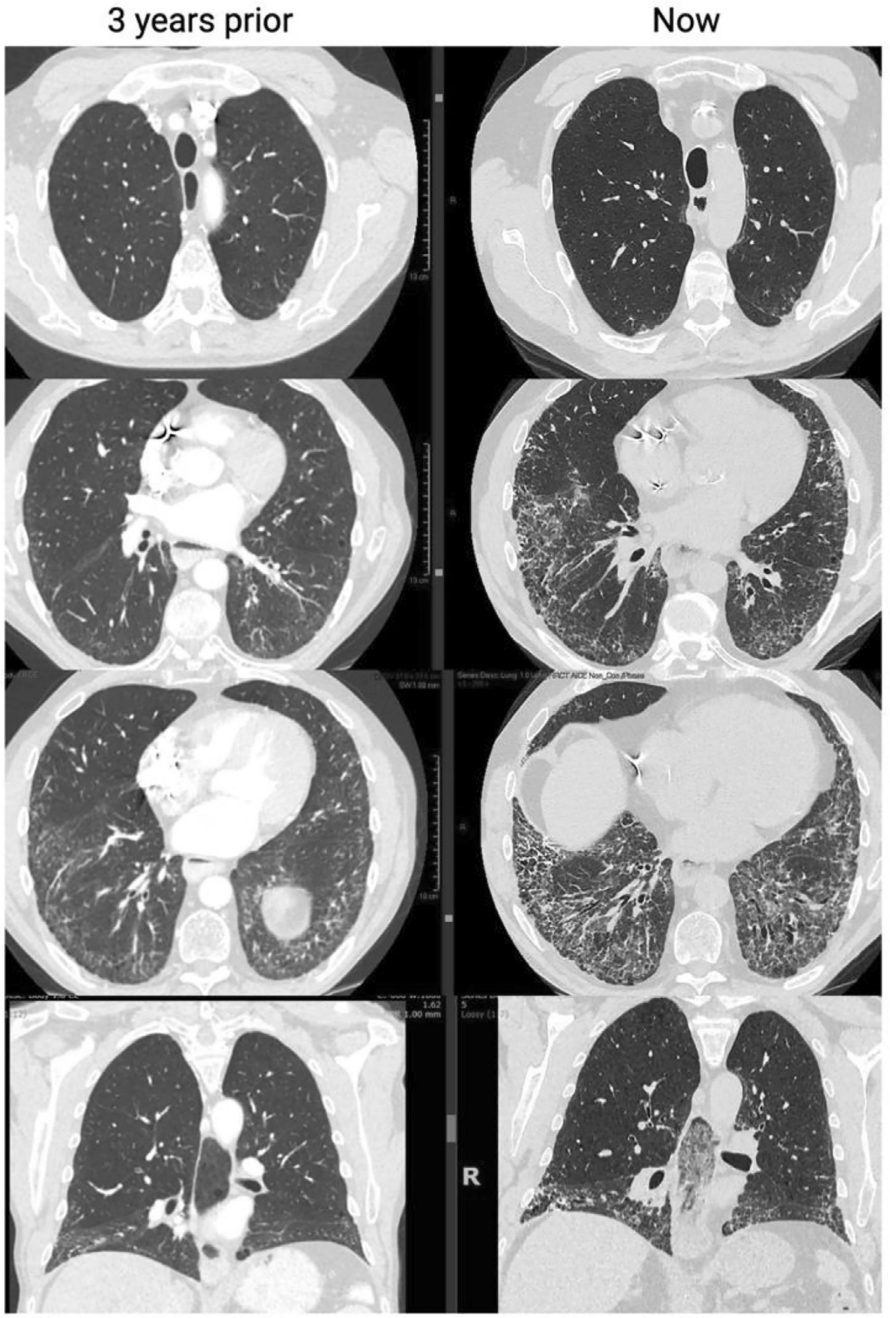
Chest high‐resolution computed tomography demonstrated progression from the scan three years prior. The scan remained in keeping in with NSIP, with ground glass opacification and reticulation alongside traction bronchiectasis and some freestanding bronchiectasis. The straight‐edge sign associated with NSIP was present. This radiologic feature is commonly reported with NSIP and less so with UIP interstitial lung disease.^27^ Diffuse dendriform pulmonary ossification, which is often associated with UIP and can also be found in NSIP, was seen.^28^ Subpleural sparing present on the previous scan was lost due to progressive fibrosis, with increased volume loss and progression of the morphology of fibrosis. Esophageal dilatation with fluid level is also demonstrated. NSIP, nonspecific interstitial pneumonia; UIP, usual interstitial pneumonia.

A repeat chest HRCT scan was performed, and images are shown in Figure 1. The fibrotic changes had significantly progressed from a previous scan three years prior. Following review of the investigations, a decision was made to treat with rituximab, a monoclonal antibody targeting CD20, leading to B cell depletion.

## Evidence for the role of B cells in SSc‐ILD

**Figure 2:**
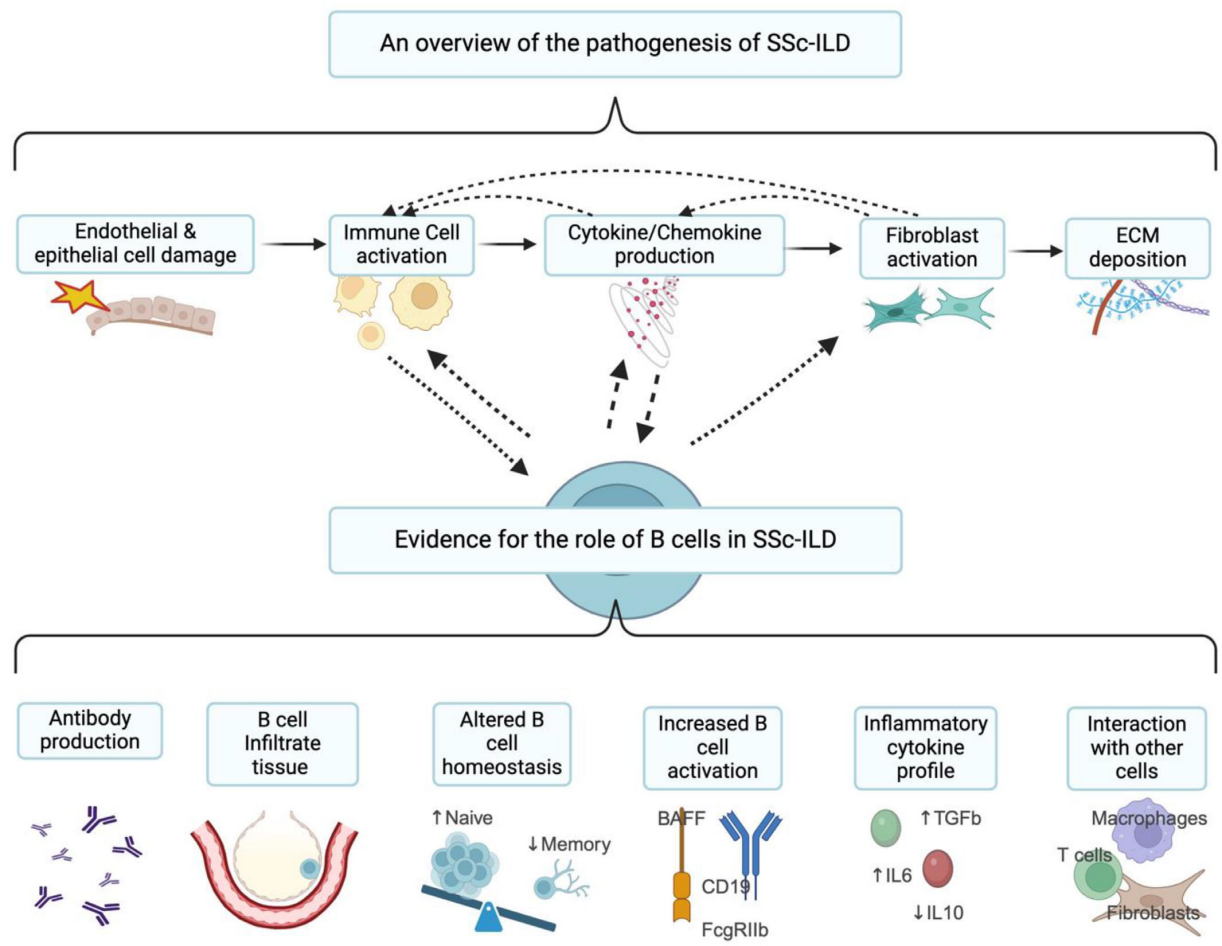
A summary of the pathogenesis of SSc‐ILD alongside the role of B cells in SSc‐ILD. ECM, extracellular matrix; ILD, interstitial lung disease; SSc, systemic sclerosis.

Dysregulated B cell function has been increasingly implicated in SSc and SSc‐ILD pathogenesis (Figure 2). Patients with SSc are consistently found to have hypergammaglobulinemia, with SSc‐specific autoantibodies found in the majority of patients.^11^, ^29^ As discussed previously, these autoantibodies strongly correlate with clinical phenotypes. However, levels of the autoantibodies have not been found to consistently be important in disease severity or trajectory, and their role in the direct pathogenesis of disease continues to be debated.^8^, ^30^, ^31^ Autoantibodies have been demonstrated to precede symptom onset.^32^ SSc‐specific antibodies (ACA, anti–RNAP III, and ATA) were the best predictive marker of progression to SSc in the Very Early Diagnosis of Systemic Sclerosis (VEDOSS) cohort.^33^

Non–ANA‐targeting autoantibodies have been described as functional autoantibodies; however, these are not routinely used in clinical practice. Antifibroblast antibodies found in patients with SSc have been demonstrated to up‐regulate profibrotic chemokines from fibroblasts.^34^ Autoantibodies targeting G protein–coupled receptors are thought to play a role in both immune response regulation in health and pathogenesis of autoimmune diseases, including SSc. For example, high concentrations of anti–endothelin 1 type A receptor and anti–angiotensin II type I receptor antibodies have been associated with a poor prognosis in SSc, and autoantibodies from patients with SSc promote vasoconstriction, fibrosis, and inflammation.^35^, ^36^

The strongest evidence of the pathogenicity of ANAs comes from ATA. Identification of several B cell immunodominant epitopes elegantly links the ATA response with development of SSc‐ILD.^37^ Immunization of mice with topoisomerase I and Freund's complete adjuvant has been demonstrated to induce skin and lung fibrosis; however, it remains unclear the contribution of ATA in this model and how ATA exerts its affect.^38^, ^39^ One proposed mechanism by which ATA directly causes fibrosis is that the autoantigen topoisomerase I, released following endothelial cell damage, binds to fibroblast surfaces, leading to recruitment of ATA. This subsequently leads to monocyte adhesion, proinflammatory cytokine release, and fibroblast activation.^40^ Within ATA‐producing ATA‐positive CD27^+^ B cells, cells with a high affinity for topoisomerase I have been found to have high production of proinflammatory cytokines, including IL‐6, and those with a low affinity for topoisomerase I have been found to have higher production of anti‐inflammatory cytokines, suggesting antibody affinity may be important in SSc pathogenesis.^41^ Anti–topoisomerase I IgM is not routinely measured, but in a cohort of ATA‐positive patients with SSc, patients with progressive disease were more often positive for anti–topoisomerase I IgM than nonprogressors, suggesting that an ongoing autoreactive B cell response is associated with disease progression.^42^ However, mixed data have been found in SSc regarding depletion of ATA levels and correlation with clinical outcomes for autologous hematopoietic stem cell transplantation (AHSCT) and B cell depletion.^43^, ^44^, ^45^, ^46^

B cell infiltration has been demonstrated in SSc, with work predominantly performed in skin due to the increased tissue accessibility compared to lungs. A small study demonstrated high expression of B lymphocyte signatures in both affected and unaffected skin in patients with early dcSSc.^47^ Another study of skin in early diffuse SSc found the majority of biopsy samples had B cell signatures (69%) along with other adaptive and innate immune cell signatures.^48^ B cell infiltration of lung tissue has been found in patients with both nonspecific interstitial pneumonia (NSIP) and usual interstitial pneumonia–pattern SSc‐ILD.^49^, ^50^ B cells may also organize into bronchus‐associated lymphoid tissue in SSc.^51^ Lymphocytosis has been found in bronchoalveolar lavage (BAL) fluid in some patients with SSc‐ILD, and a higher CD19 level has been associated with increased progression of ILD.^52^ However, the utility of BAL fluid lymphocytosis to predict progression has not been consistently demonstrated.^53^

Although absolute B cell numbers are not found to be altered in SSc, abnormal B cell homeostasis is found in disease. Increased naïve B cells and transitional B cells and reduced memory B cells have been demonstrated.^54^, ^55^ It is thought that both central and peripheral B cell tolerance are defective in SSc, resulting in autoreactive B cells involved in disease pathogenesis.^56^

B cells in SSc are thought to have a lower activation threshold, with increased presence of CD19, the positive B cell regulator, and reduction of the B cell inhibitor CD22 found in dcSSc but not lcSSc.^57^, ^58^, ^59^ BAFF and APRIL both play roles in B cell survival and activation. Inhibition of BAFF reduces skin and lung fibrosis and IL‐6–producing B cells in the bleomycin mouse model, a commonly used model of SSc.^60^ Elevated BAFF levels have been found in some studies of patients with SSc, with higher levels of both correlated with both dcSSc and ILD.^60^, ^61^ However other studies have not replicated these results.^56^

In SSc, B cells have a more proinflammatory and profibrotic phenotype. Patients with SSc have been found to have increased IL‐6–producing B cells, and although not statistically significant, a trend toward increased IL‐6–positive B cells in patients with ATA positivity and severe lung fibrosis has been seen.^62^, ^63^ Reduced production of the anti‐inflammatory cytokine IL‐10 by B cells is also reported in SSc.^64^

B cells interact directly and indirectly with other immune and stromal cells to promote fibrosis in SSc (Figure 2). B cell supernatant from patients with SSc and B cell production of antifibroblast antibody results in fibroblast activation.^34^, ^62^ T and B cell interaction has been demonstrated to be essential in ATA production in SSc.^65^ Increased circulating follicular helper T cells have been found in SSc, and these cells are important in the maturation and development of long‐lived plasma cells.^66^ B cells have also been demonstrated to induce profibrotic macrophages in a mouse model of SSc.^67^ Evidence for the role of B cells in the pathogenesis of SSc is compelling, and further evidence has also arisen from the development of treatments for SSc and SSc‐ILD, which are considered in the following section.

## Treatment options for SSc‐ILD

Several guidelines and recommendations have recently been published regarding the management of SSc and SSc‐ILD.^68^, ^69^ This reflects the changing landscape of available treatments. The recent ACR/CHEST guideline includes recommendations on screening, monitoring, and treatment of selected systemic autoimmune rheumatic diseases (SARDs) at risk of developing into ILD.^18^ In contrast, the American Thoracic Society (ATS) and the recently updated British Society for Rheumatology (BSR) guidelines were focused on SSc‐ILD and SSc, respectively.^70^ The treatment strategies in these guidelines were broadly similar, with recommendation on immunosuppression as the mainstay approach and use of antifibrotics if disease progresses despite initial management. ACR/CHEST guideline was cognizant of the variable access to therapies in different countries, and therapeutic recommendations were divided into first‐line therapies and progression after initial therapy. The ACR/CHEST guideline conditionally recommends against upfront combination of antifibrotics with MMF over MMF alone as a first‐line treatment for SARD‐ILD. In contrast, combination MMF and nintedanib was conditionally recommended in the ATS guideline. Specific comments on individual therapy are summarised^68^, ^69^, ^70^, ^71^, ^72^, ^73^, ^74^, ^75^, ^76^ in Table 2.

**Table 2:** Drugs used for the treatment of scleroderma‐associated ILD*

Drug name	Dose	Immunologic basis for action	Clinical evidence for efficacy	Adverse effects	Comments
Cyclophosphamide	Oral: 2 mg/kg daily; intravenous: 600 mg/m^2^ body surface area every 3–4 wk for a total of 6 doses	An alkylating agent that prevents replication of proliferating cells, especially bone marrow–derived leukocytes	Cyclophosphamide was associated with mean change in FVC% predicted of 2.8% at 12 mo. There was an improvement in FVC% predicted at 12 mo in a greater proportion of patients receiving cyclophosphamide compared with placebo (49.3% vs 26.4%, respectively).	Leukopenia and thrombocytopenia with increased risk of infections	Based on efficacy data, this is recommended as one of the first‐line options and for those with ILD progression in the ACR/CHEST guideline. However, the adverse effect profile led to cyclophosphamide being considered an “additional option” rather than a preferred first‐line treatment. In the ATS and BSR guidelines, this is an alternative to mycophenolate. Benefit wanes a year after cessation of cyclophosphamide.
MMF	2 g daily (maximum 3 g daily)	An inhibitor of lymphocyte guanine nucleotide synthesis that targets the enzyme inosine monophosphate dehydrogenase; some evidence that effect of MMF may be attributable, at least in part, to macrophage viability and altered alternative activation	Compared to placebo, MMF use confers a 5% difference in mean FVC% predicted of 2.8% with improvement in breathlessness (assessed with TDI score) at 24 mo. Improvement in adjusted FVC% predicted over 24 mo was observed among patients with dcSSc and early disease duration (<24 mo). There was no difference between MMF and cyclophosphamide in improvement in FVC% predicted.	Anemia, leukopenia, infection; more favorable side effect profile compared with cyclophosphamide	Recommended as a first‐line therapy in the ATS and BSR guidelines and as one of the preferred first‐line options in the ACR/CHEST guideline. MMF is better tolerated than azathioprine. Mycophenolic acid can be considered for those intolerant to MMF.
RTX	1,000 mg intravenously repeated twice at an interval of 2 wk; regimen can be repeated every 6 mo if necessary	CD20 inhibitor resulting in B cell depletion; reduction in circulating IL‐6 levels was reported at 24 wk post RTX treatment	At 24–48 wk, compared with placebo, RTX reduced the decline in FVC% predicted by 3.3%. Upfront combination MMF and RTX improves FVC over 52 wk.	Infection, including hepatitis B reactivation in particular, in combination with MMF; cytopenia	Conditionally recommended as a first‐line therapy or for those who progressed despite first‐line therapy in the ACR/CHEST guideline. Numerically fewer adverse events were reported among patients taking RTX compared to cyclophosphamide in the RECITAL study.
NTD	100–150 mg twice daily	As a tyrosine kinase inhibitor that attenuates PDGF, fibroblast‐derived growth factor, and vascular endothelial growth factor receptor signaling in fibrogenesis. There is inhibition of the profibrotic M2 phenotype via reduction of gene expression and protein synthesis of M2 cell surface markers (including CD204, CD206, and CD163) with reduction of TGFβ1 expression. NTD inhibits the mTOR‐dependent signals, leading to the expression of cytokines and matrix metalloproteinase 7 as well as B cell proliferation and IgM secretion mediators.	Compared to placebo, the adjusted annual rate of FVC decline was 40.9 mL less over 52 wk. NTD in combination with MMF provided greater numerical preservation of lung function than MMF with placebo, but this was not statistically significant.	Increased nausea, vomiting, diarrhea, weight loss, increased risk of CV events, potential increased risk of bleeding	Conditionally recommended as one of the first‐line options and when the patient meets the criteria for progression in the ACR/CHEST guideline. The ATS guideline broadens the recommendation beyond patients with progressive lung disease as well as in combination with MMF. Combination therapy with mycophenolate was not randomized in SENSCIS, and those patients receiving background mycophenolate had several differences in demographics compared with patients taking NTD alone.
Pirfenidone	Days 1–7: 267 mg 3 times per day; days 8–4: 534 mg 3 times per day; day 15 and after: 801 mg 3 times per day	Its precise mechanism of action is unclear: as an oral pyridine derivative that regulates cytokines, including TGFβ and TNF, it has antifibrotic, anti‐inflammatory, and antioxidant properties. In contrast to NTD, pirfenidone inhibits mTOR‐independent pathways in its effects on microbial antigens on B cells and their interaction with lung fibroblasts.	No statistically significant difference between treatment groups was demonstrated in the clinical studies. Small sample size did not permit subgroup analysis for the SSc‐ILD cohort.	Gastrointestinal disturbances (diarrhea, nausea, abdominal pain, weight loss), skin rash, photosensitivity	This was not recommended in current guidelines. The ATS guideline advises further research into pirfenidone alone or in combination with MMF.
Tocilizumab	162 mg subcutaneously weekly	Monoclonal antibody that acts as an IL‐6R antagonist, thus inhibiting IL‐6 activity. Reduced levels of the circulating M2‐macrophage chemokine CCL18 was reported with tocilizumab. It indirectly and directly targets B cells in SSc via inhibition of IL‐6 function. There are no studies that evaluate the effect of tocilizumab specifically on the antinuclear B cell response in SSc.	The difference in mean change from baseline to 48 wk in FVC% predicted was 6.5% less in the tocilizumab cohort.	Increased infections, hyperlipidemia, abnormal liver function	The ACR/CHEST guideline recommends this as one of the first‐line options as well as for those who progressed. Due to its shared clinical phenotype, MCTD was included in the ACR/CHEST recommendation. The ATS has a conditional recommendation. Both the ACR/CHEST and the BSR recommend this as a first‐line treatment in early dcSSc with raised markers of inflammation and ATA positivity, independent of the extent of ILD on CT. None of the guidelines recommends combination therapy with MMF.

* ACR, American College of Rheumatology; ATA, anti–topoisomerase I antibody; ATS, American Thoracic Society; BSR, British Society for Rheumatology; CHEST, American College of Chest Physicians; CT, computed tomography; CV, cardiovascular; dcSSc, diffuse cutaneous systemic sclerosis; FVC, force vital capacity; IL, interleukin; ILD, interstitial lung disease; MCTD, mixed connective tissue disease; MMF, mycophenolate mofetil; mTOR, mechanistic target of rapamycin; NTD, nintedanib; PDGF, platelet‐derived growth factor; RTX, rituximab; SSc, systemic sclerosis; TDI, transition dyspnea index; TGF, transforming growth factor; TNF, tumor necrosis factor.

MMF is recommended as a first‐line therapy, with evidence from the Scleroderma Lung Study II showing equivalence of MMF to cyclophosphamide but an improved side effect profile with MMF.^76^ Cyclophosphamide can be used as a rescue or alternative to MMF. Given the significant reduction in ILD progression, MMF was recommended as a first‐line therapy in both the ATS and the BSR guidelines (Table 2). Prednisolone, however, is not recommended in SSc‐ILD due to increased risk of scleroderma renal crisis. The ACR/CHEST guideline strongly recommends against steroid use, and both the ATS and the ACR/CHEST guidelines advise that the daily dose should not exceed the equivalent of 15 mg of prednisone. The risk and benefit of steroid use need to be carefully considered in the context of coexisting inflammatory disease, such as myositis or arthritis, and alternative use of immunosuppression should be considered if the patient is at risk of developing renal crisis.

Tocilizumab, the monoclonal antibody against IL‐6, has been demonstrated to stabilize FVC in a randomized controlled trial (RCT) of SSc‐ILD in early diffuse inflammatory SSc.^75^, ^77^ More recently, rituximab in the RECITAL and DESIRES RCTs has also been found to stabilize lung function.^78^, ^79^, ^80^ The EVER‐ILD trial of NSIP‐pattern ILD, which included a small cohort of patients with SSc‐ILD, suggested that combination rituximab and MMF was superior to MMF alone in improvement of FVC.^81^ The benefit of combination therapy on FVC response was not demonstrated to be sustained at 12 months; however, progression‐free survival was better with combination therapy, and, of note, the majority of patients did not receive maintenance rituximab therapy.^82^ In the BSR guideline, rituximab and tocilizumab are recommended as an addition to MMF as rescue therapy. Tocilizumab is suggested as a possible first‐line therapy in patients with early dcSSc and raised levels of markers of inflammation and particularly those who are ATA positive.^70^ The ACR/CHEST guideline also recommends rituximab and tocilizumab as possible alternative first‐line options to MMF.^69^ A recent retrospective analysis suggests that ATA‐positive patients have a particular benefit from tocilizumab therapy compared to ATA‐negative patients.^83^ Interestingly our data also suggest that a more diverse cohort of patients continue to benefit from rituximab and tocilizumab therapy than were included in clinical trials, including those who remain refractory to standard immunosuppression irrespective of disease duration, inflammatory response, and skin subset.

Robust data from clinical trials support use of AHSCT in specialist centers for dcSSc, and this can be used for SSc‐ILD if disease progresses despite initial management; however, careful patient selection must be performed.^84^ Severe internal organ involvement precludes AHSCT due to high treatment‐related toxicity. Further evidence is required regarding both the outcomes with early use of AHSCT, which the ongoing UPSIDE trial is addressing, and comparisons of AHSCT with early combination immunosuppression.^85^

Nintedanib has been demonstrated to be effective in attenuating lung function decline in SSc‐ILD in patients with a progressive fibrosing phenotype.^86^, ^87^ The ACR/CHEST and BSR guidelines preferentially recommend nintedanib as an additional therapy after immunosuppression; however, the ACR/CHEST guideline acknowledges it could be a first‐line therapy in certain patients, and the BSR guideline recommends that it could be used in upfront combination with MMF for patients with extensive ILD at disease onset.^70^

## Evidence for the B cell therapies

The success of B cell therapies, including rituximab, in improving outcomes in SSc‐ILD provides support for the key role of B cells in the SSc pathogenesis, and an increasing number of studies that target B cells are currently in progress in SSc‐ILD (Table 3). Rapid peripheral B cell depletion at two weeks after rituximab treatment has been correlated with treatment response for SSc‐ILD, and a small retrospective study found that complete B cell depletion was associated with improved response to rituximab in connective tissue disease associated ILD (CTD‐ILD).^91^, ^92^

**Table 3:** B cell therapies in SSc*

Target	Agent	Study design	Outcome	Reference	ClinicalTrials.gov identifier
Completed B cell–directed therapies in SSc					
CD20	RTX	RCT (phase 2/3)	RTX improved mRSS and lung function in SSc	Ebata et al^79^	NCT04274257
CD20	RTX	RCT (phase 2)	No statistical improvement but potential treatment for SSc‐PAH	Zamanian et al^88^	NCT01086540
CD20	RTX	RCT (phase 2/3)	RTX increased FVC at 24 wk for CTD‐ILD, including SSc, but was not superior to cyclophosphamide. Improvement in mRSS. Steroid‐sparing effect was noted.	Maher et al^78^	NCT0182926
CD20	RTX	RCT (phase 3)	Upfront RTX and MMF combination stabilized lung function at 6 mo	Mankikian et al^81^	NCT02990286
CD19	Inebilizumab	RCT (phase 1)	Single escalating dose of MEDI‐551 was well tolerated and safe	Schiopu et al^89^	NCT00946699
BAFF	Belimumab	RCT (phase 2)	No significant effect on mRSS at 52 wk with belimumab–MMF compared to MMF alone	Gordon et al^90^	NCT01670565
Enrolling trials investigating B cell–targeted therapies for SSc					
BAFF and CD20	Belimumab and RTX	RCT (phase 2)	Ongoing; primary outcome evaluating change in the ACR rCRISS at 12 mo	–	NCT03844061
BAFF	Belimumab with standard therapy	RCT (Bliss‐ILD phase 2/3)	Ongoing; primary outcome on lung function at week 52	–	NCT05878717
BAFF and IL‐17	Tibulizumab	RCT (TibuSURE phase 2)	Not recruiting yet; primary outcome on skin at week 24	–	NCT06843239
BAFF receptor	Ianalumab	RCT (phase 2)	Recruiting; primary outcome evaluating rCRISS response at week 52	–	NCT06470048
CD20	Divozilimab	RCT (phase 3)	Not recruiting; primary outcome on skin at week 24	–	NCT05726630

* ACR, American College of Rheumatology; CTD, connective tissue disease; FVC, force vital capacity; IL, interleukin; ILD, interstitial lung disease; MMF, mycophenolate mofetil; mRSS, modified Rodnan skin score; PAH, pulmonary arterial hypertension; rCRISS, revised Composite Response Index in Systemic Sclerosis; RCT, randomized controlled trial; RTX, rituximab; SSc, systemic sclerosis.

Rituximab, however, does not effectively deplete all B cells. For example, B cell precursors and plasmablasts that lack CD20 expression are not targeted, and memory B cells in lesional tissue can be relatively protected.^93^ It has been considered whether this failure to deplete all B cells and lack of change in autoantibody titers after rituximab treatment result in failure or incomplete treatment response.^45^, ^46^

AHSCT results in a shift of B cell homeostasis, with an increase in naïve B cells and a reduction in memory B cells. The B cell compartment appears to shift to a more Breg cell phenotype, with an increase in IL‐10 levels and a reduction in IL‐6–producing B cells.^94^ However, the high treatment‐related toxicity of AHSCT has led to the consideration of CD19 chimeric antigen receptor (CAR) T cells as an alternative cellular therapy that may have an advantageous safety profile.^95^, ^96^

CAR T cell therapy that targets CD19 potentially may overcome the limitations of CD20‐targeted strategies, including rituximab, and may target lesional pathogenic antibodies, conferring a deep depletive effect. This is supported by individual case studies and case series of autologous CD19 CAR T cell strategies, which have shown improvement in FVC (median 195 mL) and a 4% median reduction in ILD extent on HRCT, in addition to improvement in the mRSS.^97^ Using the CRISPR/Cas9 strategy with a healthy donor–derived allogeneic CD19 CAR T cell product in two patients with dcSSc, improvement in ILD without graft‐versus‐host disease–related adverse effects was recently reported.^98^ Bispecific autoantigen T cell–engaging antibodies are a new class of “off‐the‐shelf” B cell–depletion approaches that do not require personalized manufacturing. Blinatumomab was reported to improve the mRSS and result in profound B cell depletion in a patient with progressive dcSSc.^99^ Similar preclinical progress on engineering CARs for expression on Treg cells to recognize particular self‐antigens is under evaluation. It is envisaged that the pace of innovation in CAR T cell development for autoantibody‐mediated diseases, including SSc, will continue to accelerate (ClinicalTrials.gov identifiers NCT06400303, NCT06347718, and NCT06328777).

As discussed previously, data regarding BAFF in SSc are mixed. Elevated BAFF levels have been found following treatment with rituximab in SSc, and BAFF levels have been associated with flares following rituximab treatment in systemic lupus erythematosus.^73^, ^100^ A small trial of belimumab, a monoclonal antibody targeting BAFF, demonstrated no significant improvement in skin when administered in addition to MMF for early dcSSc, and a phase 2/3 trial of belimumab for SSc‐ILD is ongoing.^90^, ^101^ Ianalumab is an anti‐BAFF receptor monoclonal antibody that has a dual mechanism of action with blockade of BAFF receptor–mediated signaling and B cell depletion by enhanced antibody‐dependent cellular cytotoxicity.^102^ A phase 2 clinical trial of ianalumab in early dcSSc has recently begun^103^ (Table 3).

Although IL‐6 is produced by a range of cells, in SSc, it has been suggested^62^ that B cells may be the predominant source of increased levels of IL‐6. Rituximab has been demonstrated to reduce IL‐6 levels in SSc, and the interaction of tocilizumab with B cells is likely important in the mechanism of action of this treatment.^73^

## Infection risk with immunosuppression

Concerns regarding the use of rituximab and other biologics, especially in combination with other immunosuppressants, often revolve around risk of infection. Infection risk may also increase over time with hypogammaglobulinemia from B cell depletion antibody therapy.^104^, ^105^

In the RECITAL trial, there was reduced steroid exposure in patients with CTD‐ILD taking rituximab compared to cyclophosphamide, and infection rates were similar across groups; however, rituximab was not used in combination with other immunosuppression.^78^ In the EVER‐ILD trial, in which rituximab was given in combination with 2 g daily of MMF, infection rates were higher in the combination therapy group compared to those receiving MMF alone; however, increased infections were predominantly nonserious viral infections.^81^

An increased risk of severe infection, hospitalization, and death from COVID‐19 in patients receiving rituximab for immune‐mediated disease compared to the general population has been found.^106^ It has also been shown that there is an increased risk of COVID‐19 infection with rituximab in patients with inflammatory rheumatic diseases receiving rituximab compared to those receiving other disease‐modifying antirheumatic drugs and nonrituximab biologic agents.^106^, ^107^ This risk is of greater concern with additional risk factors for severe COVID‐19 infection present, such as ILD.^108^

Rituximab treatment also results in a reduction in the humoral response to the COVID‐19 vaccine, although the functional T cell response is unaltered to the messenger RNA vaccine.^109^ A single‐center study of patients with autoimmune disease treated with rituximab in the United Kingdom found that full vaccination significantly reduced rates of COVID‐19 infection, and although breakthrough infection rates were high after vaccination, most cases were mild in fully vaccinated individuals).^110^ Hypogammaglobulinemia before rituximab treatment was found to be associated with moderate and severe COVID‐19 infection. However, of note in this study, 74% (261 of 352) received their first vaccine dose more than six months after rituximab treatment. Current ACR guidelines recommend that COVID‐19 vaccination timing be carefully considered in relation to rituximab dosing, either using CD19 B cell measurements to time vaccination or by giving booster doses two to four weeks before the next anticipated dose.^111^

Due to the B cell–targeting action of rituximab, there is no evidence of increased risk of tuberculosis with rituximab.^112^ Risk of hepatitis B reactivation with rituximab treatment is clear, and screening is recommended before rituximab initiation.^113^ *Pneumocystis jirovecii* pneumonia (PJP) prevention is also recommended for patients with ILD taking rituximab.

## Case follow‐up

**Figure 3:**
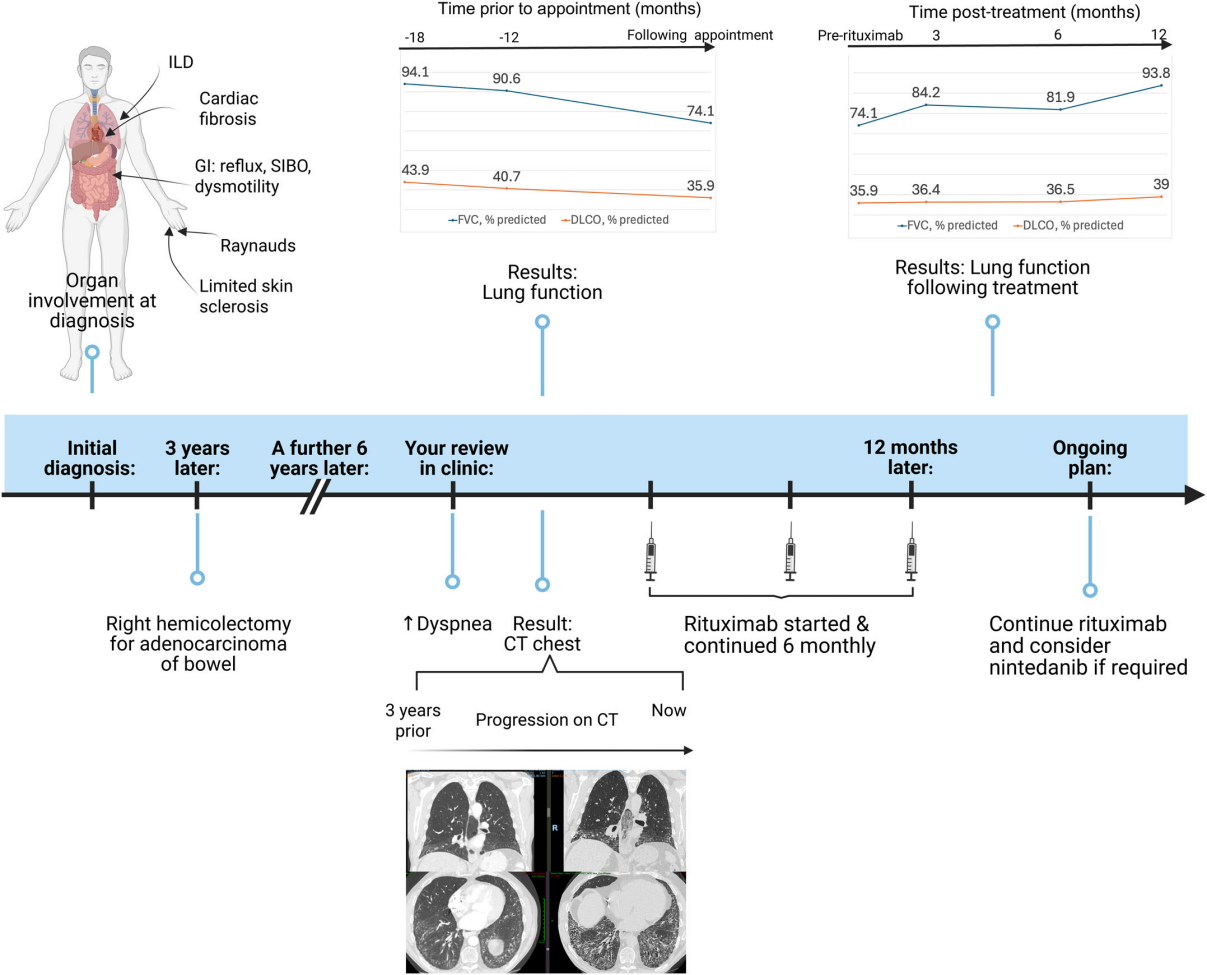
A pictorial summary of the case. CT, computed tomography; DLCO, diffusing capacity for carbon monoxide; FVC, forced vital capacity; GI, gastrointestinal; ILD, interstitial lung disease; SIBO, small instestinal bacterial overgrowth. Color figure can be viewed in the online issue, which is available at http://onlinelibrary.wiley.com/doi/10.1002/art.43326/abstract.

Following vaccination against COVID‐19 and after commencing PJP prophylaxis, the patient was treated with rituximab (two doses of 1 g with dosing interval of two weeks) and continued with MMF 2 g daily. He was given further rituximab treatment at the same dose six months later. No infections requiring antibiotic therapy occurred in the first year following rituximab initiation. A repeat lung function test was performed for monitoring after rituximab treatment, and results are shown in Supplemental Table 1. A decision was made to continue rituximab therapy every six months and keep nintedanib in reserve if SSc‐ILD progresses further (Figure 3).

Rituximab treatment appears to have stabilized SSc‐ILD in this patient with long‐standing SSc and normal markers of inflammation. This case highlights the diverse range of patients with progressive SSc‐ILD, for whom additional biologic therapy can be considered, as well as the need to be cognizant of non‐ATA high‐risk SSc‐ILD antibodies. Although continued rituximab treatment is planned, the durability of long‐term response beyond the observed benefit with initial treatment is also unknown.

## Conclusions

Future studies will determine whether CD19‐targeting CAR T cells or the CD20 antibody obinutuzumab may be more effective or have an unacceptable infection risk compared to rituximab. The clear message is that B cells may have a key role in SSc‐ILD, and further development of both cell‐ and antibody‐based B cell–depleting therapies are justified.

## AUTHOR CONTRIBUTIONS

All authors contributed to at least one of the following manuscript preparation roles: conceptualization AND/OR methodology, software, investigation, formal analysis, data curation, visualization, and validation AND drafting or reviewing/editing the final draft. As corresponding author, Dr Denton confirms that all authors have provided the final approval of the version to be published and takes responsibility for the affirmations regarding article submission (eg, not under consideration by another journal), the integrity of the data presented, and the statements regarding compliance with institutional review board/Declaration of Helsinki requirements.

## Supporting information


**Disclosure form**.

**Table S1:** Lung function trajectory

Figure S1:

## References

[1] LeRoy EC , Black C , Fleischmajer R , et al. Scleroderma (systemic sclerosis): classification, subsets and pathogenesis. J Rheumatol 1988;15(2):202–205. 3361530

[2] Elhai M , Meune C , Boubaya M , et al; EUSTAR group. Mapping and predicting mortality from systemic sclerosis. Ann Rheum Dis 2017;76(11):1897–1905. 28835464 10.1136/annrheumdis-2017-211448

[3] Steen VD , Medsger TA . Changes in causes of death in systemic sclerosis, 1972‐2002. Ann Rheum Dis 2007;66(7):940–944. 17329309 10.1136/ard.2006.066068 PMC1955114

[4] Harrison NK , Myers AR , Corrin B , et al. Structural features of interstitial lung disease in systemic sclerosis. Am Rev Respir Dis 1991;144(3 Pt 1):706–713. 1892314 10.1164/ajrccm/144.3_Pt_1.706

[5] Truchetet ME , Brembilla NC , Chizzolini C . Current concepts on the pathogenesis of systemic sclerosis. Clin Rev Allergy Immunol 2023;64(3):262–283. 34487318 10.1007/s12016-021-08889-8 PMC10167130

[6] Liu X , Dai K , Zhang X , et al. Multiple fibroblast subtypes contribute to matrix deposition in pulmonary fibrosis. Am J Respir Cell Mol Biol 2023;69(1):45–56. 36927333 10.1165/rcmb.2022-0292OC PMC10324043

[7] Fanning GC , Welsh KI , Bunn C , et al. HLA associations in three mutually exclusive autoantibody subgroups in UK systemic sclerosis patients. Br J Rheumatol 1998;37(2):201–207. 9569077 10.1093/rheumatology/37.2.201

[8] Nihtyanova SI , Sari A , Harvey JC , et al. Using autoantibodies and cutaneous subset to develop outcome‐based disease classification in systemic sclerosis. Arthritis Rheumatol 2020;72(3):465–476. 31682743 10.1002/art.41153

[9] Perelas A , Silver RM , Arrossi AV , et al. Systemic sclerosis‐associated interstitial lung disease. Lancet Respir Med 2020;8(3):304–320. 32113575 10.1016/S2213-2600(19)30480-1

[10] De Lauretis A , Sestini P , Pantelidis P , et al. Serum interleukin 6 is predictive of early functional decline and mortality in interstitial lung disease associated with systemic sclerosis. J Rheumatol 2013;40(4):435–446. 23378460 10.3899/jrheum.120725

[11] Steen VD . Autoantibodies in systemic sclerosis. Semin Arthritis Rheum 2005;35(1):35–42. 16084222 10.1016/j.semarthrit.2005.03.005

[12] Acosta‐Herrera M , Kerick M , Lopéz‐Isac E , et al; International SSc Group; Australian Scleroderma Interest Group (ASIG). Comprehensive analysis of the major histocompatibility complex in systemic sclerosis identifies differential HLA associations by clinical and serological subtypes. Ann Rheum Dis 2021;80(8):1040–1047. 34096881 10.1136/annrheumdis-2021-219884 PMC8292594

[13] Nihtyanova SI , Denton CP . Autoantibodies as predictive tools in systemic sclerosis. Nat Rev Rheumatol 2010;6(2):112–116. 20125179 10.1038/nrrheum.2009.238

[14] Yamashita Y , Yamano Y , Muro Y , et al. Clinical significance of anti‐NOR90 antibodies in systemic sclerosis and idiopathic interstitial pneumonia. Rheumatology (Oxford) 2022;61(4):1709–1716. 34282441 10.1093/rheumatology/keab575

[15] Fritzler MJ , Hudson M , Choi MY , et al; Canadian Scleroderma Research Group. Bicaudal D2 is a novel autoantibody target in systemic sclerosis that shares a key epitope with CENP‐A but has a distinct clinical phenotype. Autoimmun Rev 2018;17(3):267–275. 29369808 10.1016/j.autrev.2018.01.006

[16] Adler BL , Boin F , Wolters PJ , et al. Autoantibodies targeting telomere‐associated proteins in systemic sclerosis. Ann Rheum Dis 2021;80(7):912–919. 33495152 10.1136/annrheumdis-2020-218918 PMC8217217

[17] Kuwana M , Gil‐Vila A , Selva‐O'Callaghan A . Role of autoantibodies in the diagnosis and prognosis of interstitial lung disease in autoimmune rheumatic disorders. Ther Adv Musculoskelet Dis 2021;13:1759720X211032457. 10.1177/1759720X211032457 PMC8320553 34377160

[18] Johnson SR , Bernstein EJ , Bolster MB , et al. 2023 American College of Rheumatology (ACR)/American College of Chest Physicians (CHEST) guideline for the screening and monitoring of interstitial lung disease in people with systemic autoimmune rheumatic diseases. Arthritis Rheumatol 2024;76(8):1201–1213. 38973714 10.1002/art.42860 PMC12646464

[19] Fertig N , Domsic RT , Rodriguez‐Reyna T , et al. Anti‐U11/U12 RNP antibodies in systemic sclerosis: a new serologic marker associated with pulmonary fibrosis. Arthritis Rheum 2009;61(7):958–965. 19565553 10.1002/art.24586 PMC2739404

[20] Fritzler MJ , Bentow C , Beretta L , et al. Anti‐U11/U12 antibodies as a rare but important biomarker in patients with systemic sclerosis: a narrative review. Diagnostics (Basel) 2023;13(7):1257. 37046475 10.3390/diagnostics13071257 PMC10093660

[21] Callejas‐Moraga EL , Guillén‐Del‐Castillo A , Perurena‐Prieto J , et al. Anti‐RNPC‐3 antibody predicts poor prognosis in patients with interstitial lung disease associated to systemic sclerosis. Rheumatology (Oxford) 2021;61(1):154–162. 33742673 10.1093/rheumatology/keab279

[22] Shah AA , Xu G , Rosen A , et al. Brief report: anti‐RNPC‐3 antibodies as a marker of cancer‐associated scleroderma. Arthritis Rheumatol 2017;69(6):1306–1312. 28217959 10.1002/art.40065 PMC5449218

[23] Will CL , Schneider C , Hossbach M , et al. The human 18S U11/U12 snRNP contains a set of novel proteins not found in the U2‐dependent spliceosome. RNA 2004;10(6):929–941. 15146077 10.1261/rna.7320604 PMC1370585

[24] Gilliam AC , Steitz JA . Rare scleroderma autoantibodies to the U11 small nuclear ribonucleoprotein and to the trimethylguanosine cap of U small nuclear RNAs. Proc Natl Acad Sci USA 1993;90(14):6781–6785. 8341699 10.1073/pnas.90.14.6781 PMC47016

[25] McMahan ZH , Domsic RT , Zhu L , et al. Anti‐RNPC‐3 (U11/U12) antibodies in systemic sclerosis in patients with moderate‐to‐severe gastrointestinal dysmotility. Arthritis Care Res (Hoboken) 2019;71(9):1164–1170. 30242973 10.1002/acr.23763 PMC6430701

[26] Mahler M , Roup F , Bentow C , et al. Anti‐RNPC‐3 antibodies are associated with nuclear speckled immunofluorescence pattern and enriched in triple negative systemic sclerosis patients [abstract]. Arthritis Rheumatol 2019;71(suppl 10). https://acrabstracts.org/abstract/anti‐rnpc‐3‐antibodies‐are‐associated‐with‐nuclear‐speckled‐immunofluorescence‐pattern‐and‐enriched‐in‐triple‐negative‐systemic‐sclerosis‐patients/

[27] Zhan X , Koelsch T , Montner SM , et al. Differentiating usual interstitial pneumonia from nonspecific interstitial pneumonia using high‐resolution computed tomography: the “straight‐edge sign”. J Thorac Imaging 2018;33(4):266–270. 29683868 10.1097/RTI.0000000000000328

[28] Egashira R , Jacob J , Kokosi MA , et al. Diffuse pulmonary ossification in fibrosing interstitial lung diseases: prevalence and associations. Radiology 2017;284(1):255–263. 28182861 10.1148/radiol.2017152419

[29] Fleischmajer R , Perlish JS , Reeves JRT . Cellular infiltrates in scleroderma skin. Arthritis Rheum 1977;20(4):975–984. 861067 10.1002/art.1780200410

[30] Hu PQ , Fertig N , Medsger TA Jr , et al. Correlation of serum anti‐DNA topoisomerase I antibody levels with disease severity and activity in systemic sclerosis. Arthritis Rheum 2003;48(5):1363–1373. 12746909 10.1002/art.10977

[31] Tramposch HD , Smith CD , Senecal JL , et al. A long‐term longitudinal study of anticentromere antibodies. Arthritis Rheum 1984;27(2):121–124. 6607733 10.1002/art.1780270201

[32] Burbelo PD , Gordon SM , Waldman M , et al. Autoantibodies are present before the clinical diagnosis of systemic sclerosis. PLoS One 2019;14(3):e0214202. 30913258 10.1371/journal.pone.0214202 PMC6435159

[33] Minier T , Guiducci S , Bellando‐Randone S , et al; EUSTAR co‐workers; EUSTAR co‐workers . Preliminary analysis of the very early diagnosis of systemic sclerosis (VEDOSS) EUSTAR multicentre study: evidence for puffy fingers as a pivotal sign for suspicion of systemic sclerosis. Ann Rheum Dis 2014;73(12):2087–2093. 23940211 10.1136/annrheumdis-2013-203716

[34] Fineschi S , Goffin L , Rezzonico R , et al. Antifibroblast antibodies in systemic sclerosis induce fibroblasts to produce profibrotic chemokines, with partial exploitation of toll‐like receptor 4. Arthritis Rheum 2008;58(12):3913–3923. 19035500 10.1002/art.24049

[35] Kill A , Tabeling C , Undeutsch R , et al. Autoantibodies to angiotensin and endothelin receptors in systemic sclerosis induce cellular and systemic events associated with disease pathogenesis. Arthritis Res Ther 2014;16(1):R29. 24472528 10.1186/ar4457 PMC3978438

[36] Akbarzadeh R , Müller A , Humrich JY , et al. When natural antibodies become pathogenic: autoantibodies targeted against G protein‐coupled receptors in the pathogenesis of systemic sclerosis. Front Immunol 2023;14:1213804. 37359516 10.3389/fimmu.2023.1213804 PMC10285309

[37] Rizou C , Ioannidis JPA , Panou‐Pomonis E , et al. B‐cell epitope mapping of DNA topoisomerase I defines epitopes strongly associated with pulmonary fibrosis in systemic sclerosis. Am J Respir Cell Mol Biol 2000;22(3):344–351. 10696071 10.1165/ajrcmb.22.3.3850

[38] Liem SIE , Neppelenbroek S , Fehres CM , et al. Autoreactive B cell responses targeting nuclear antigens in systemic sclerosis: implications for disease pathogenesis. Semin Arthritis Rheum 2023;58:152136. 36403538 10.1016/j.semarthrit.2022.152136

[39] Yoshizaki A , Yanaba K , Ogawa A , et al. Immunization with DNA topoisomerase I and Freund's complete adjuvant induces skin and lung fibrosis and autoimmunity via interleukin‐6 signaling. Arthritis Rheum 2011;63(11):3575–3585. 21792823 10.1002/art.30539

[40] Senécal JL , Hoa S , Yang R , et al. Pathogenic roles of autoantibodies in systemic sclerosis: current understandings in pathogenesis. J Scleroderma Relat Disord 2020;5(2):103–129. 35382028 10.1177/2397198319870667 PMC8922609

[41] Fukasawa T , Yoshizaki A , Ebata S , et al. Single‐cell‐level protein analysis revealing the roles of autoantigen‐reactive B lymphocytes in autoimmune disease and the murine model. eLife 2021;10:e67209. 34854378 10.7554/eLife.67209 PMC8639144

[42] Boonstra M , Bakker JA , Grummels A , et al. Association of anti‐topoisomerase I antibodies of the IgM isotype with disease progression in anti‐topoisomerase I‐positive systemic sclerosis. Arthritis Rheumatol 2020;72(11):1897–1904. 32840062 10.1002/art.41403 PMC7702063

[43] Farge D , Henegar C , Carmagnat M , et al. Analysis of immune reconstitution after autologous bone marrow transplantation in systemic sclerosis. Arthritis Rheum 2005;52(5):1555–1563. 15880600 10.1002/art.21036

[44] Henes J , Glaeser L , Kötter I , et al. Analysis of anti‐topoisomerase I antibodies in patients with systemic sclerosis before and after autologous stem cell transplantation. Rheumatology (Oxford) 2017;56(3):451–456. 27940597 10.1093/rheumatology/kew319

[45] Bonroy C , Smith V , Deschepper E , et al. Specific antinuclear antibody level changes after B cell depletion therapy in systemic sclerosis are associated with improvement of skin thickening. J Rheumatol 2016;43(3):681. 10.3899/jrheum.150105.C1 26932995

[46] Lafyatis R , Kissin E , York M , et al. B cell depletion with rituximab in patients with diffuse cutaneous systemic sclerosis. Arthritis Rheum 2009;60(2):578–583. 19180481 10.1002/art.24249 PMC2637937

[47] Whitfield ML , Finlay DR , Murray JI , et al. Systemic and cell type‐specific gene expression patterns in scleroderma skin. Proc Natl Acad Sci USA 2003;100(21):12319–12324. 14530402 10.1073/pnas.1635114100 PMC218756

[48] Skaug B , Khanna D , Swindell WR , et al. Global skin gene expression analysis of early diffuse cutaneous systemic sclerosis shows a prominent innate and adaptive inflammatory profile. Ann Rheum Dis 2020;79(3):379–386. 31767698 10.1136/annrheumdis-2019-215894 PMC7386329

[49] Lafyatis R , O'Hara C , Feghali‐Bostwick CA , et al. B cell infiltration in systemic sclerosis‐associated interstitial lung disease. Arthritis Rheum 2007;56(9):3167–3168. 17763433 10.1002/art.22847

[50] Arvelo Castro L , Van Keulen V , Ali M , et al. B‐cell infiltration varies among patients with interstitial lung diseases [abstract]. Eur Respir J 2016;48(suppl 60):PA3099.

[51] Popper H , Stacher‐Priehse E , Brcic L , et al. Lung fibrosis in autoimmune diseases and hypersensitivity: how to separate these from idiopathic pulmonary fibrosis. Rheumatol Int 2022;42(8):1321–1330. 34605934 10.1007/s00296-021-05002-2 PMC9287245

[52] De Santis M , Bosello SL , Peluso G , et al. Bronchoalveolar lavage fluid and progression of scleroderma interstitial lung disease. Clin Respir J 2012;6(1):9–17. 21801327 10.1111/j.1752-699X.2010.00228.x

[53] Goh NSL , Veeraraghavan S , Desai SR , et al. Bronchoalveolar lavage cellular profiles in patients with systemic sclerosis‐associated interstitial lung disease are not predictive of disease progression. Arthritis Rheum 2007;56(6):2005–2012. 17530640 10.1002/art.22696

[54] Sato S , Fujimoto M , Hasegawa M , et al. Altered blood B lymphocyte homeostasis in systemic sclerosis: expanded naive B cells and diminished but activated memory B cells. Arthritis Rheum 2004;50(6):1918–1927. 15188368 10.1002/art.20274

[55] Forestier A , Guerrier T , Jouvray M , et al. Altered B lymphocyte homeostasis and functions in systemic sclerosis. Autoimmun Rev 2018;17(3):244–255. 29343447 10.1016/j.autrev.2017.10.015

[56] Glauzy S , Olson B , May CK , et al. Defective early B cell tolerance checkpoints in patients with systemic sclerosis allow the production of self antigen‐specific clones. Arthritis Rheumatol 2022;74(2):307–317. 34279059 10.1002/art.41927 PMC8766600

[57] Soto L , Ferrier A , Aravena O , et al. Systemic sclerosis patients present alterations in the expression of molecules involved in B‐cell regulation. Front Immunol 2015;6:496. 26483788 10.3389/fimmu.2015.00496 PMC4586944

[58] Sato S , Hasegawa M , Fujimoto M , et al. Quantitative genetic variation in CD19 expression correlates with autoimmunity. J Immunol 2000;165(11):6635–6643. 11086109 10.4049/jimmunol.165.11.6635

[59] Melissaropoulos K , Liossis SN . Decreased CD22 expression and intracellular signaling aberrations in B cells of patients with systemic sclerosis. Rheumatol Int 2018;38(7):1225–1234. 29869007 10.1007/s00296-018-4076-3

[60] Matsushita T , Hasegawa M , Yanaba K , et al. Elevated serum BAFF levels in patients with systemic sclerosis: enhanced BAFF signaling in systemic sclerosis B lymphocytes. Arthritis Rheum 2006;54(1):192–201. 16385515 10.1002/art.21526

[61] Matsushita T , Fujimoto M , Hasegawa M , et al. Elevated serum APRIL levels in patients with systemic sclerosis: distinct profiles of systemic sclerosis categorized by APRIL and BAFF. J Rheumatol 2007;34(10):2056–2062. 17896803

[62] Dumoitier N , Chaigne B , Régent A , et al. Scleroderma peripheral B lymphocytes secrete interleukin‐6 and transforming growth factor β and activate fibroblasts. Arthritis Rheumatol 2017;69(5):1078–1089. 27992693 10.1002/art.40016

[63] Taher TE , Ong VH , Bystrom J , et al. Association of defective regulation of autoreactive interleukin‐6‐producing transitional B lymphocytes with disease in patients with systemic sclerosis. Arthritis Rheumatol 2018;70(3):450–461. 29193892 10.1002/art.40390

[64] Matsushita T , Hamaguchi Y , Hasegawa M , et al. Decreased levels of regulatory B cells in patients with systemic sclerosis: association with autoantibody production and disease activity. Rheumatology (Oxford) 2016;55(2):263–267. 26350483 10.1093/rheumatology/kev331

[65] Kuwana M , Medsger TA Jr , Wright TMT . T and B cell collaboration is essential for the autoantibody response to DNA topoisomerase I in systemic sclerosis. J Immunol 1995;155(5):2703–2714. 7650398

[66] Ricard L , Jachiet V , Malard F , et al. Circulating follicular helper T cells are increased in systemic sclerosis and promote plasmablast differentiation through the IL‐21 pathway which can be inhibited by ruxolitinib. Ann Rheum Dis 2019;78(4):539–550. 30760472 10.1136/annrheumdis-2018-214382

[67] Numajiri H , Kuzumi A , Fukasawa T , et al. B cell depletion inhibits fibrosis via suppression of profibrotic macrophage differentiation in a mouse model of systemic sclerosis. Arthritis Rheumatol 2021;73(11):2086–2095. 33955200 10.1002/art.41798

[68] Raghu G , Montesi SB , Silver RM , et al. Treatment of systemic sclerosis‐associated interstitial lung disease: evidence‐based recommendations. an official American Thoracic Society clinical practice guideline. Am J Respir Crit Care Med 2024;209(2):137–152. 37772985 10.1164/rccm.202306-1113ST PMC10806429

[69] Johnson SR , Bernstein EJ , Bolster MB , et al. 2023 American College of Rheumatology (ACR)/American College of Chest Physicians (CHEST) guideline for the treatment of interstitial lung disease in people with systemic autoimmune rheumatic diseases. Arthritis Rheumatol 2024;76(8):1182–1200. 38978310 10.1002/art.42861 PMC12646471

[70] Denton CP , De Lorenzis E , Roblin E , et al. The 2024 British Society for Rheumatology guideline for management of systemic sclerosis. Rheumatology (Oxford) 2024;63(11):2956–2975. 39255973 10.1093/rheumatology/keae394 PMC11534099

[71] Ali MF , Egan AM , Shaughnessy GF , et al. Antifibrotics modify B‐cell‐induced fibroblast migration and activation in patients with idiopathic pulmonary fibrosis. Am J Respir Cell Mol Biol 2021;64(6):722–733. 33689587 10.1165/rcmb.2020-0387OC PMC8456878

[72] Morris EA , Parvizi R , Orzechowski NM , et al. Mycophenolate mofetil directly modulates myeloid viability and pro‐fibrotic activation of human macrophages. Rheumatology (Oxford) 2025;64(5):3125–3133. 39312626 10.1093/rheumatology/keae517 PMC12048068

[73] Bosello S , De Santis M , Lama G , et al. B cell depletion in diffuse progressive systemic sclerosis: safety, skin score modification and IL‐6 modulation in an up to thirty‐six months follow‐up open‐label trial. Arthritis Res Ther 2010;12(2):R54. 20338043 10.1186/ar2965 PMC2888203

[74] Soldano S , Smith V , Montagna P , et al. Nintedanib downregulates the profibrotic M2 phenotype in cultured monocyte‐derived macrophages obtained from systemic sclerosis patients affected by interstitial lung disease. Arthritis Res Ther 2024;26(1):74. 38509595 10.1186/s13075-024-03308-7 PMC10953168

[75] Khanna D , Denton CP , Jahreis A , et al. Safety and efficacy of subcutaneous tocilizumab in adults with systemic sclerosis (faSScinate): a phase 2, randomised, controlled trial. Lancet 2016;387(10038):2630–2640. 27156934 10.1016/S0140-6736(16)00232-4

[76] Tashkin DP , Roth MD , Clements PJ , et al; Sclerodema Lung Study II Investigators. Mycophenolate mofetil versus oral cyclophosphamide in scleroderma‐related interstitial lung disease (SLS II): a randomised controlled, double‐blind, parallel group trial. Lancet Respir Med 2016;4(9):708–719. 27469583 10.1016/S2213-2600(16)30152-7 PMC5014629

[77] Khanna D , Lin CJF , Furst DE , et al; focuSSced investigators . Tocilizumab in systemic sclerosis: a randomised, double‐blind, placebo‐controlled, phase 3 trial. Lancet Respir Med 2020;8(10):963–974. 32866440 10.1016/S2213-2600(20)30318-0

[78] Maher TM , Tudor VA , Saunders P , et al; RECITAL Investigators . Rituximab versus intravenous cyclophosphamide in patients with connective tissue disease‐associated interstitial lung disease in the UK (RECITAL): a double‐blind, double‐dummy, randomised, controlled, phase 2b trial. Lancet Respir Med 2023;11(1):45–54. 36375479 10.1016/S2213-2600(22)00359-9

[79] Ebata S , Yoshizaki A , Oba K , et al. Safety and efficacy of rituximab in systemic sclerosis (DESIRES): a double‐blind, investigator‐initiated, randomised, placebo‐controlled trial. Lancet Rheumatol 2021;3(7):e489–e497. 38279402 10.1016/S2665-9913(21)00107-7

[80] Ebata S , Yoshizaki A , Oba K , et al. Safety and efficacy of rituximab in systemic sclerosis (DESIRES): open‐label extension of a double‐blind, investigators‐initiated, randomised, placebo‐controlled trial. Lancet Rheumatol 2022;4(8):e546–e555. 38294008 10.1016/S2665-9913(22)00131-X

[81] Mankikian J , Caille A , Reynaud‐Gaubert M , et al; EVER‐ILD investigators and the OrphaLung network. Rituximab and mycophenolate mofetil combination in patients with interstitial lung disease (EVER‐ILD): a double‐blind, randomised, placebo‐controlled trial. Eur Respir J 2023;61(6):2202071. 37230499 10.1183/13993003.02071-2022

[82] Mansy L , Caille A , Reynaud‐Gaubert M , et al; EVER‐ILD investigators and the OrphaLung network . Rituximab and mycophenolate mofetil in interstitial lung disease (EVER‐ILD): 1‐year follow‐up results of a randomised controlled trial. Eur Respir J 2024;64(3):2401368. 39231630 10.1183/13993003.01368-2024

[83] Goldman NR , Nihtyanova SI , Beesley CF , et al. Tocilizumab and rituximab for systemic sclerosis interstitial lung disease: a real‐world cohort analysis. Rheumatology (Oxford) Published online January 3, 2025. 10.1093/rheumatology/keaf006 PMC12695047 39752322

[84] Bruera S , Sidanmat H , Molony DA , et al. Stem cell transplantation for systemic sclerosis. Cochrane Database Syst Rev. 2022;(7):CD011819. 35904231 10.1002/14651858.CD011819.pub2 PMC9336163

[85] Spierings J , van Rhenen A , Welsing PMW , et al. A randomised, open‐label trial to assess the optimal treatment strategy in early diffuse cutaneous systemic sclerosis: the UPSIDE study protocol. BMJ Open 2021;11(3):e044483. 10.1136/bmjopen-2020-044483 PMC7978271 33737437

[86] Flaherty KR , Wells AU , Cottin V , et al; INBUILD Trial Investigators . Nintedanib in progressive fibrosing interstitial lung diseases. N Engl J Med 2019;381(18):1718–1727. 31566307 10.1056/NEJMoa1908681

[87] Distler O , Highland KB , Gahlemann M , et al; SENSCIS Trial Investigators . Nintedanib for systemic sclerosis‐associated interstitial lung disease. N Engl J Med 2019;380(26):2518–2528. 31112379 10.1056/NEJMoa1903076

[88] Zamanian RT , Badesch D , Chung L , et al. Safety and efficacy of B‐cell depletion with rituximab for the treatment of systemic sclerosis‐associated pulmonary arterial hypertension: a multicenter, double‐blind, randomized, placebo‐controlled trial. Am J Respir Crit Care Med 2021;204(2):209–221. 33651671 10.1164/rccm.202009-3481OC PMC8650794

[89] Schiopu E , Chatterjee S , Hsu V , et al. Safety and tolerability of an anti‐CD19 monoclonal antibody, MEDI‐551, in subjects with systemic sclerosis: a phase I, randomized, placebo‐controlled, escalating single‐dose study. Arthritis Res Ther 2016;18(1):131. 27267753 10.1186/s13075-016-1021-2 PMC4895815

[90] Gordon JK , Martyanov V , Franks JM , et al. Belimumab for the treatment of early diffuse systemic sclerosis: results of a randomized, double‐blind, placebo‐controlled, pilot trial. Arthritis Rheumatol 2018;70(2):308–316. 29073351 10.1002/art.40358 PMC6590997

[91] Ebata S , Yoshizaki A , Fukasawa T , et al. Percentage of residual B cells after 2 weeks of rituximab treatment predicts the improvement of systemic sclerosis‐associated interstitial lung disease. J Dermatol 2022;49(1):179–183. 34661314 10.1111/1346-8138.16206

[92] Al Tabaa O , Ghossan R , Combier A , et al. Sustained complete B cell depletion is associated with rituximab efficacy in connective tissue disorders‐associated interstitial lung disease. RMD Open 2023;9(1):e002832. 36754549 10.1136/rmdopen-2022-002832 PMC9923347

[93] Hofmann K , Clauder AK , Manz RA . Targeting B cells and plasma cells in autoimmune diseases Front Immunol 2018;9:835. 29740441 10.3389/fimmu.2018.00835 PMC5924791

[94] Lima‐Júnior JR , Arruda LCM , Gonçalves MS , et al. Autologous haematopoietic stem cell transplantation restores the suppressive capacity of regulatory B cells in systemic sclerosis patients. Rheumatology (Oxford) 2021;60(12):5538–5548. 33724344 10.1093/rheumatology/keab257

[95] Bergmann C , Müller F , Distler JHW , et al. Treatment of a patient with severe systemic sclerosis (SSc) using CD19‐targeted CAR T cells. Ann Rheum Dis 2023;82(8):1117–1120. 37147112 10.1136/ard-2023-223952 PMC10359520

[96] Merkt W , Freitag M , Claus M , et al. Third‐generation CD19.CAR‐T cell‐containing combination therapy in Scl70+ systemic sclerosis. Ann Rheum Dis 2024;83(4):543–546. 38135464 10.1136/ard-2023-225174 PMC10958299

[97] Auth J , Müller F , Völkl S , et al. CD19‐targeting CAR T‐cell therapy in patients with diffuse systemic sclerosis: a case series. Lancet Rheumatol 2025;7(2):e83–e93. 39542003 10.1016/S2665-9913(24)00282-0

[98] Wang X , Wu X , Tan B , et al. Allogeneic CD19‐targeted CAR‐T therapy in patients with severe myositis and systemic sclerosis. Cell 2024;187(18):4890–4904.e9. 39013470 10.1016/j.cell.2024.06.027

[99] Subklewe M , Magno G , Gebhardt C , et al. Application of blinatumomab, a bispecific anti‐CD3/CD19 T‐cell engager, in treating severe systemic sclerosis: a case study. Eur J Cancer 2024;204:114071. 38691878 10.1016/j.ejca.2024.114071

[100] Carter LM , Isenberg DA , Ehrenstein MR . Elevated serum BAFF levels are associated with rising anti‐double‐stranded DNA antibody levels and disease flare following B cell depletion therapy in systemic lupus erythematosus. Arthritis Rheum 2013;65(10):2672–2679. 23839909 10.1002/art.38074

[101] Denton CP , Spiera R , Jörg D , et al. Belimumab for the treatment of interstitial lung disease associated with systemic sclerosis: a phase 2/3, randomised, double‐blind, placebo‐controlled trial [abstract]. Ann Rheum Dis 2023;82(suppl 1):1668.

[102] Dörner T , Posch MG , Li Y , et al. Treatment of primary Sjögren's syndrome with ianalumab (VAY736) targeting B cells by BAFF receptor blockade coupled with enhanced, antibody‐dependent cellular cytotoxicity. Ann Rheum Dis 2019;78(5):641–647. 30826774 10.1136/annrheumdis-2018-214720

[103] A clinical study to evaluate ianalumab in participants with diffuse cutaneous systemic sclerosis. ClinicalTrials.gov identifier: NCT06470048. Updated August 7, 2024. Accessed September 20, 2024. https://clinicaltrials.gov/study/NCT06470048

[104] van Vollenhoven RF , Emery P , Bingham CO III , et al. Long‐term safety of rituximab in rheumatoid arthritis: 9.5‐year follow‐up of the global clinical trial programme with a focus on adverse events of interest in RA patients. Ann Rheum Dis 2013;72(9):1496–1502. 23136242 10.1136/annrheumdis-2012-201956 PMC3756452

[105] Nie Y , Zhang N , Li J , et al. Hypogammaglobulinemia and infection events in patients with autoimmune diseases treated with rituximab: 10 years real‐life experience. J Clin Immunol 2024;44(8):179. 39150626 10.1007/s10875-024-01773-y

[106] Patel NJ , D'Silva KM , Hsu TYT , et al. Coronavirus disease 2019 outcomes among recipients of Anti‐CD20 monoclonal antibodies for immune‐mediated diseases: a comparative cohort study. ACR Open Rheumatol 2022;4(3):238–246. 34890478 10.1002/acr2.11386 PMC8916578

[107] Felten R , Duret PM , Bauer E , et al. B‐cell targeted therapy is associated with severe COVID‐19 among patients with inflammatory arthritides: a 1‐year multicentre study in 1116 successive patients receiving intravenous biologics. Ann Rheum Dis 2022;81(1):143–145. 34556483 10.1136/annrheumdis-2021-220549

[108] Gao YD , Ding M , Dong X , et al. Risk factors for severe and critically ill COVID‐19 patients: a review Allergy 2021;76(2):428–455. 33185910 10.1111/all.14657

[109] Bitoun S , Henry J , Desjardins D , et al. Rituximab impairs B cell response but not T cell response to COVID‐19 vaccine in autoimmune diseases. Arthritis Rheumatol 2022;74(6):927–933. 34962357 10.1002/art.42058 PMC9011892

[110] Md Yusof MY , Arnold J , Saleem B , et al. Breakthrough SARS‐CoV‐2 infections and prediction of moderate‐to‐severe outcomes during rituximab therapy in patients with rheumatic and musculoskeletal diseases in the UK: a single‐centre cohort study. Lancet Rheumatol 2023;5(2):e88–e98. 36712951 10.1016/S2665-9913(23)00004-8 PMC9873269

[111] Curtis JR , Johnson SR , Anthony DD , et al. American College of Rheumatology guidance for COVID‐19 vaccination in patients with rheumatic and musculoskeletal diseases: version 5. Arthritis Rheumatol 2023;75(1):E1–E16. 36345691 10.1002/art.42372 PMC9878068

[112] Cantini F , Nannini C , Niccoli L , et al. Risk of tuberculosis reactivation in patients with rheumatoid arthritis, ankylosing spondylitis, and psoriatic arthritis receiving non‐anti‐TNF‐targeted biologics. Mediators Inflamm 2017;2017:8909834. 28659665 10.1155/2017/8909834 PMC5474286

[113] Varley CD , Winthrop KL . Long‐term safety of rituximab (risks of viral and opportunistic infections). Curr Rheumatol Rep 2021;23(9):74. 34269903 10.1007/s11926-021-01037-3 PMC8284038

