# Supplementary material for: Case‐Based Immunology: B Cells and Systemic Sclerosis Interstitial Lung Disease
Source: Arthritis Rheumatol. 2025 Dec 2;78(3):566–81. doi: 10.1002/art.43326 (PMC12991924; doi:10.1002/art.43326)
Supplement: Supplementary file 2 — Table S1: Lung function trajectory [file ART-78-566-s001.docx]

**Table 1**: Lung function trajectory

|  | **FVC, %Pred ( L)** | **DLCO ,%Pred (mmol/min/kPa)** | **FEV1% (L)** |
| --- | --- | --- | --- |
| **Jan-15** | 92.7 (3.78) | 61.3 (5.08) | 85.4 (3.23) |
| **Oct-16** | 100.8 (4.05) | 49.9 (4.08) | 86.7 (3.31) |
| **Jan-17** | 105.3 (4.22) | 50.6 (4.14) | 88.2 (3.43) |
| **Dec-17** | 97.3(3.87) | 48.3 (3.93) | 87 (3.30) |
| **Mar-18** | 97.3 (3.87) | 45.9 (3.73) | 87.5 (3.30) |
| **Jun-19** | 98.2 (3.90) | 55.2 (4.45) | 86.8 (3.28) |
| **Nov-20** | 94.1 (3.74) | 43.9 (3.66) | 84.8 (3.26) |
| **May-21** | 90.6 (3.68) | 40.7 (3.41) | 85.3 (3.28) |
| **May-22** | 74.1 (3.01) | 35.9 (3.01) | 71.4 (1.91) |
| **Nov-22** | 84.2 (3.21) | 36.4 (3.05) | 73.6 (2.14) |
| **Mar-23** | 81.9 (3.10) | 36.5 (3.06) | 69.1 (2.04) |
| **Nov-23** | 93.8(3.54) | 39 (3.27) | 66.6 (2.23) |

* FEV1, forced expiratory volume in 1 second; FVC, forced vital capacity; DLCO, diffusing capacity for carbon
