# Supplementary figures and images for: Case‐Based Immunology: B Cells and Systemic Sclerosis Interstitial Lung Disease
Source: Arthritis Rheumatol. 2025 Dec 2;78(3):566–81. doi: 10.1002/art.43326 (PMC12991924; doi:10.1002/art.43326)

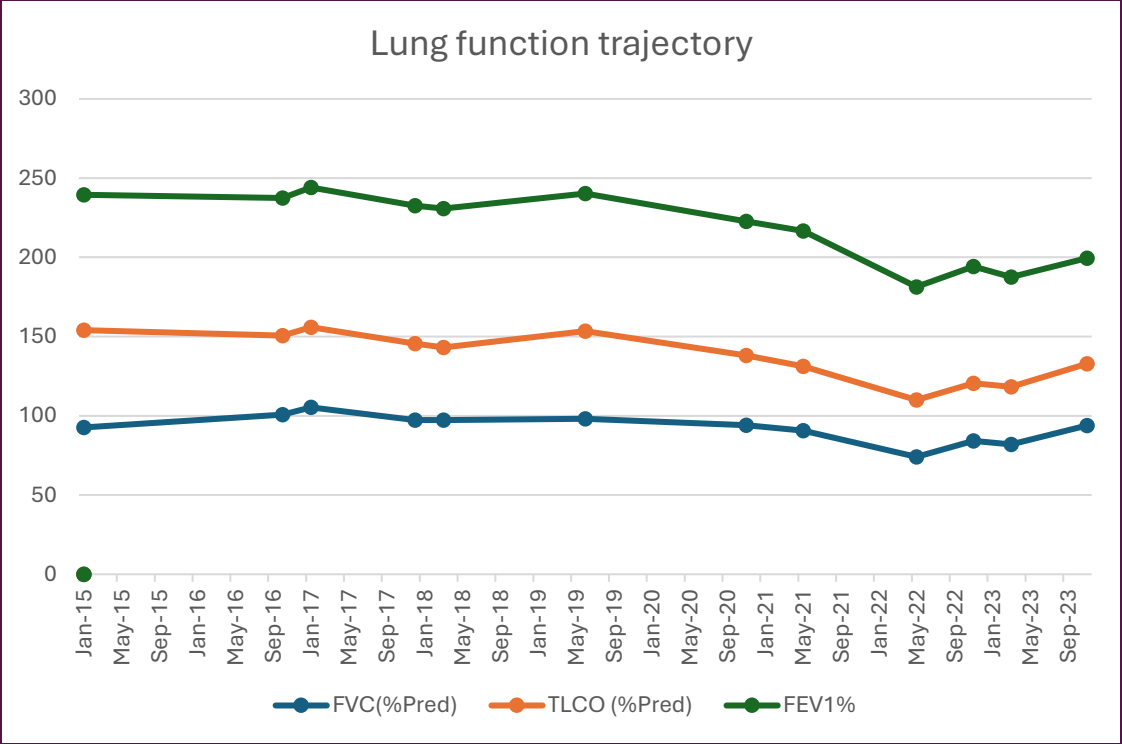

Supplement: Supplementary file 3 — Figure S1: [file ART-78-566-s003.pdf]
